# Bio-Based PBT–DLA Copolyester as an Alternative Compatibilizer of PP/PBT Blends

**DOI:** 10.3390/polym11091421

**Published:** 2019-08-29

**Authors:** Wojciech Ignaczak, Peter Sobolewski, Miroslawa El Fray

**Affiliations:** West Pomeranian University of Technology, Szczecin, Faculty of Chemical Technology and Engineering, Polymer Institute, Al. Piastów 45, 71-311 Szczecin, Poland

**Keywords:** polymer blends and alloys, PP/PBT, thermoplastic elastomers, compatibilization, fatty acid

## Abstract

The aim of this work was to assess whether synthesized random copolyester, poly(butylene terephthalate-*r*-butylene dilinoleate) (PBT–DLA), containing bio-based components, can effectively compatibilize polypropylene/poly(butylene terephthalate) (PP/PBT) blends. For comparison, a commercial petrochemical triblock copolymer, poly(styrene-*b*-ethylene/butylene-*b*-styrene) (SEBS) was used. The chemical structure and block distribution of PBT–DLA was determined using nuclear magnetic resonance spectroscopy and gel permeation chromatography. PP/PBT blends with different mass ratios were prepared via twin-screw extrusion with 5 wt% of each compatibilizer. Thermogravimetric analysis, differential scanning calorimetry, and dynamic mechanical analysis were used to assess changes in phase structure of PP/PBT blends. Static tensile testing demonstrated marked improvement in elongation at break, to ~18% and ~21% for PBT–DLA and SEBS, respectively. Importantly, the morphology of PP/PBT blends compatibilized with PBT–DLA copolymer showed that it is able to act as interphase modifier, being preferentially located at the interface. Therefore, we conclude that by using polycondensation and monomers from renewable resources, it is possible to obtain copolymers that efficiently modify blend miscibility, offering an alternative to widely used, rubber-like petrochemical styrene compatibilizers.

## 1. Introduction

Different types of polymers can be used to develop polymeric blends, by simple physical mixing, as new materials with selectively enhanced properties. Various physical and mechanical properties of a polymer blend can be modified by varying the chemical composition. However, most polymer blends are immiscible; therefore, their properties are not only a function of their composition but also depend crucially on the miscibility of the components. Further, many pairs of polymers are not only immiscible, but also incompatible. This is mainly due to lack of specific interactions between blend constituents, differences in structures of the components, and significant differences in viscosities. As a result, they exhibit high interfacial tension, leading to rough phase structure, poor adhesion at the interface, and, as a result, poor mechanical properties [1,2]. 

Polymer blends have been studied extensively since the 1970s, with several comprehensive reviews available discussing various compatibilization strategies [3,4]. Further, recent environmental and sustainability concerns have led to interest in polymer blending and compatibilization as part of recycling strategies of mixed plastic waste [5]. Of particular interest is the use of compatibilizers for the preparation of recycled blends of poly(ethylene terephthalate) (PET) and polyolefins [6,7,8,9]. Likewise, compatibilization of blends of recycled poly(butylene terephthalate) (rPBT) from scrapped cars and neat polypropylene (PP) has also been investigated [10].

Compatibilization of immiscible and incompatible polymer blends can be achieved using various methods, including nonreactive and reactive compatibilizers [11,12]. Compatibilizers can be efficiently used to enhance polymer blend preparation, and a great deal of research has been devoted to the possibility of the optimization of immiscible blends through the use of compatibilizers such as polypropylene grafted with maleic anhydride (PP-*g*-MA), poly(styrene-ethylene/butylene-styrene) (SEBS) copolymers, phenoxy ethyl vinyl ether grafted polypropylene (PP-*g*-PHEVE), etc. [13,14,15,16,17,18,19,20]. In situ blend preparation, by a reactive compatibilization process, has also been investigated [21,22]. Compatibilization with the use of a third component can significantly improve the mechanical properties of the blend by the reduction of dispersed phase domain size and enhancement of interlaminar adhesion. For example, a high compatibilizing effect, resulting in improved miscibility, was obtained using styrene and maleic anhydride monomer melt-grafted polypropylene in immiscible and incompatible PP/polyamide 6 (PA6) ternary blends [23]. Similarly, an improvement in the morphology of PP/PET blends has been reported using compatibilizers based on PP grafted with maleic anhydride (PP-*g*-MA) [24]. In recent years, some authors have also highlighted that glycidyl methacrylate (GMA) modified SEBS [25] and styrene-b-(ethylene-*co*-propylene) (SEP) can be successfully used as compatibilizers of PP/PET blends [26], yielding remarkable reduction of phase dispersion and approx. two times greater impact resistance. The effect of enhanced miscibility was observed in markedly increased melt viscosity, which was ascribed to the occurrence of chemical reactions between the epoxide groups of GMA and the carboxyl/hydroxyl end groups of PET during melt mixing. Another group showed that the addition of ethylene–glycidyl methacrylate copolymers yields PET/PP blends with well-dispersed morphology and improved elongation at break [9]. For compatibilization of the same blend, triblock copolymer SEBS grafted with maleic anhydride has also been shown to be useful for the enhancement of mechanical properties [27]. Overall, comparative research of the compatibilization process of PP/PET blends with different compatibilizers [28], taking into account mechanical and thermal properties, as well as morphology, classifies them in following order, in terms of their effectiveness: 1. SEBS-*g*-MA, PP-*g*-MA + TPO; 2. LLDPE-*g*-MA; 3. PP-*g*-MA.

In addition to the chemical composition, the architecture and sequence arrangement of the copolymer to be used as compatibilizer are also important parameters that influence the solubility of a copolymer and, thus, the ability to compatibilize the polymer blend [29]. The block/segment distribution of a linear copolymer can vary from alternating to blocky to random, which plays an important role in its ability to enhance blend miscibility at the interface. Experimental and theoretical studies have confirmed that block copolymers are able to form different geometrical forms at the interface, starting from cylindrical for diblock copolymers and more isotropic with increasing number of blocks (crossing the interface many times) [30,31,32]. Furthermore, as the copolymer becomes more random or alternating, it can cover a more tangible part of the interface. Additionally, some theories [32,33,34,35,36] predict that the more times a copolymer molecule crosses the interface, the more effective it can be as an interfacial modifier, by forming “stiches”—connections or joints—bonding both phases together. Different copolymer structures, such as random [32,33], diblock [35,36], multiblock [37,38], alternating [39], and graft [36], have been tested as compatibilizers, but the results do not provide conclusive insight into the role of copolymer architecture on the quality of interfacial adhesion. This is most likely because other parameters, including the chemical composition of copolymers, molecular weight, and blend composition, are not comparable between the various studies.

In this work, we compared the compatibilization of immiscible PP/PBT blends using two copolymers featuring thermoplastic elastomer behavior: (1) commonly-used and commercially available triblock of poly(styrene-*b*-ethylene/butylene-*b*-styrene) (SEBS) grafted with maleic anhydride, which we have previously studied [40], and (2) synthesized poly(butylene terephthalate-*r*-butylene dilinoleate) (PBT–DLA) with random architecture (30/70 wt% ratio). The motivation for this research was the potential replacement of commercially used compatibilizers, mostly based on styrene components, with copolymers synthesized using monomers from renewable resources. Dilinoleic acid (a dimerized fatty acid derivative) is derived from vegetable oils [41], and both comonomers, dimethyl terephthalate and 1,4-butanediol, are also obtainable from biomass feedstock [42]. Thus, this copolymer can be 100% bio-based—to our knowledge, we are the only group investigating such compatibilizers. This is increasingly important, because bio-based approaches towards obtaining both of the components of the blend (PP and PBT) are also being actively explored [42,43]. It is our hypothesis that PBT–DLA is well suited to compatibilize PP/PBT blends, because of the presence of both PBT hard segments, entangling with the PBT homopolymer, and alkyl chains of the butylene dilinoleate soft segments, interacting favorably with the PP homopolymer. In order to build upon our previous work with PBT–DLA copolymer with 50/50 wt% ratio [44] and improve the compatibilization of the blends, here we used a larger proportion of butylene dilinoleate soft segments, and we performed a detailed analysis of the PBT–DLA copolymer structure, in order to understand how this copolymer is located at the interface. In addition to mechanical testing, thermal analysis techniques, common tools used for polymer characterization [45], and SEM microscopy were used to better understand the phenomena occurring at the interfacial region of PP/PBT blends differing in mass ratio of respective components.

## 2. Materials and Methods

### 2.1. Material Selection

Nucleated polypropylene (PP) (Moplen HP 2409), used as the PP blend component, was obtained from Basell Polyolefins (Płock, Poland). Neat poly(butylene terephthalate) (PBT) (Celanex 1600A) was obtained from Ticona Engineering Polymers (Frankfurt, Germany). Both materials have been developed for extrusion, thermoforming, and film applications. Blend components were selected based on the mass melt flow index (MFI) at the blend processing temperature (230 °C) (MFI_PP_:MFI_PBT_, 2.5:3.0 g/10 min). Commercially available triblock copolymer, poly(styrene-*b*-ethylene/butylene-*b*-styrene) grafted with maleic anhydride (SEBS-*g*-MAH) (Kraton FG 1901GT), used as compatibilizer, was obtained from Kraton LLC Polymers (Houston, Texas, USA). The styrene (hard segment) content in the SEBS block copolymer was 30 wt%. The random copolymer PBT–DLA, consisting of hard segments of butylene terephthalate (30 wt%) and soft segments of butylene dilinolate (an ester of dimerized fatty acid, here dilinoleic acid, and 1,4-butanediol), was synthesized as previously described [44,46], in two stages: (1) transesterification of dimethyl terephthalate with 1,4-butanediol and (2) polycondensation with addition of dilinoleic acid (dimerized fatty acid, Pripol 1009, Croda, Gouda, Netherlands). We synthesized PBT–DLA with the same content of hard segments/blocks (30 wt%) as the SEBS copolymer, in order to obtain better insight into the effect of molecular architecture on the compatibilization mechanism.

### 2.2. Characterization of Compatibilizers

SEBS copolymer (one of the first thermoplastic elastomers developed), according to the literature [21,47], is a triblock copolymer without diblock fractions; selected physicochemical properties are summarized in Table 1. 

We performed detailed structural characterization of the PBT–DLA copolymer synthesized in our lab using nuclear magnetic resonance (NMR) spectroscopy. NMR spectra were obtained using a TM Bruker DPX 400 MHz spectrometer (Bruker, Billerica, Massachusetts, USA) with tetramethylsilane (TMS) as an internal reference. A sample of dried polymer was dissolved in CDCl_3_ (50 mg/mL), and 128 scans and 2048 scans were collected for ^1^H NMR and ^13^C NMR, respectively, at room temperature and a 1 s relaxation time. 

### 2.3. Melt Blending

PP/PBT blends with different mass ratios (60/40; 50/50; 40/60 wt%) and 5 wt% of compatibilizer (SEBS or PBT–DLA) were prepared using twin-screw extrusion. The amount of compatibilizer was chosen based on our previous work [40]. Prior to extrusion, all polymers were dried for 24 h at 60 °C, and then held in vacuo at 40 °C for 48 h to fully eliminate moisture. The melt blending was carried out using a 16 mm co-rotating screw extruder Thermo Electron Prism Eurolab 16 (ThermoFisher Scientific, Waltham, Massachusetts, USA) with L/D = 40, barrel temperature ranging from 220 to 230 °C, and nozzle temperature set to 230 °C. Screw rotation speed as well as dosing rate were determined in order to achieve an outlet pressure of 6 to 8 bar. After extrusion, polymer blends were cut into 3 mm pellets, dried for 24 h at 60 °C, and then held in vacuo at 40 °C for 48 h to fully eliminate moisture. Test specimens were injection molded according to the PN-EN ISO 527-2 standard using a BOY 32 (Dr. Boy GmbH & Co. K, Neustadt-Fernthal, Germany) injection molding machine with L/D = 22. The injection pressure was the same for all polymer blends: 50 bar, with a holding phase of 40 bar.

### 2.4. Characterization of Polymer Blends

Injection-molded test specimens (S1 dog-bone shaped) were conditioned for 48 h at room temperature, and then their morphology, thermal, and mechanical properties were characterized.

Thermogravimetric analysis (TGA), as a function of increasing temperature, was used to analyze the thermal stability of polymer blends. For thermogravimetric analysis, a TGA Q500 (TA Instruments, New Castle, Delaware, USA) was used with platinum crucibles. A heating rate of 2 °C/min over a temperature range of 25–600 °C under dry air, using <15 mg of sample, was applied. For each blend, the experiment was repeated twice.

To examine phase change behavior, differential scanning calorimetry (DSC) was performed, using Q100 DSC (TA Instruments, New Castle, Delaware, USA) apparatus. Samples were weighed (~10 mg) into aluminum pans and hermetically sealed before the analysis from −60 to 260 °C, with a heating/cooling rate of 10 °C/min. 

Q800 DMA (TA Instruments, New Castle, Delaware, USA) apparatus operating in a dual cantilever mode was used to determine the storage modulus (G’), loss modulus (G’’), and the tangent of the phase angle (tan δ). The glass transition temperature (*T*_g_) was taken as the maximum of tan δ. The relaxation spectrum was scanned from −70 to 150 °C, at a frequency of 1 Hz, and a heating rate of 3 °C/min. 

Static tensile tests were performed using an Instron 3366 machine (Instron, Norwood, Massachusetts, USA). Injection-molded test specimens (dog-bones S1) were conditioned for 48 h at room temperature prior to mechanical tests. The strain rate for Young’s Modulus measurement was 1 mm/min. Elongation at break was evaluated at a crosshead speed of 50 mm/min. A video extensometer was used to measure the displacement. Additionally, Charpy notched tests were performed to measure the impact properties of PP/PBT blends using 0,5 J Zwick/Roel Hammer (Zwick GmbH & Co. KG, Ulm, Germany) at room temperature, with A-type notch samples. For each blend, at least 7 samples were measured.

High-resolution scanning electron microscopy (HRSEM, JSM-6100, JEOL Ltd., Tokyo, Japan) was used to examine the blend morphology (before and after compatibilization). The specimens were fractured in liquid nitrogen, and the fracture surface was examined. All the specimens were coated with a thin gold layer prior to SEM analysis, in order to avoid electrostatic charging. Micrographs were taken in high-vacuum mode, at an acceleration voltage of 7 kV and a magnification of 1000–5000 times.

## 3. Results and Discussion

### 3.1. Characterization of PBT–DLA Structure

**Figure 1:**
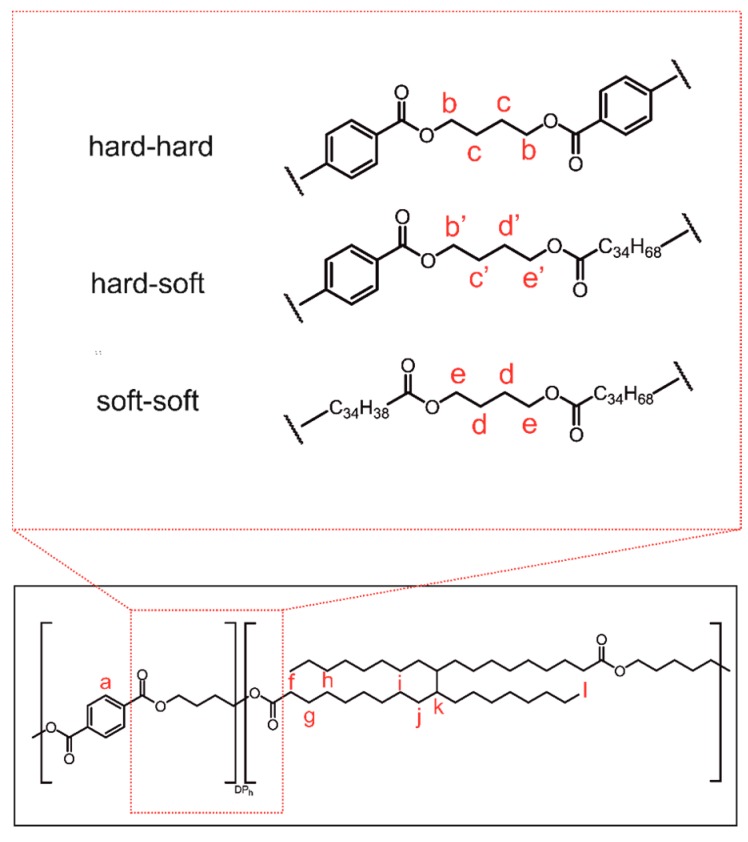
Chemical structure of poly(butylene terephthalate-*r*-butylene dilinoleate) (PBT–DLA) copolymer. The inset (**top**) presents the possible arrangements and combinations of adjacent segments, hard: butylene terephthalate, soft: butylene dilinoleate. Characteristic protons discussed in the text are marked in red. DP_h_—theoretical degree of polycondensation of hard segments = 1.21.

**Figure 2:**
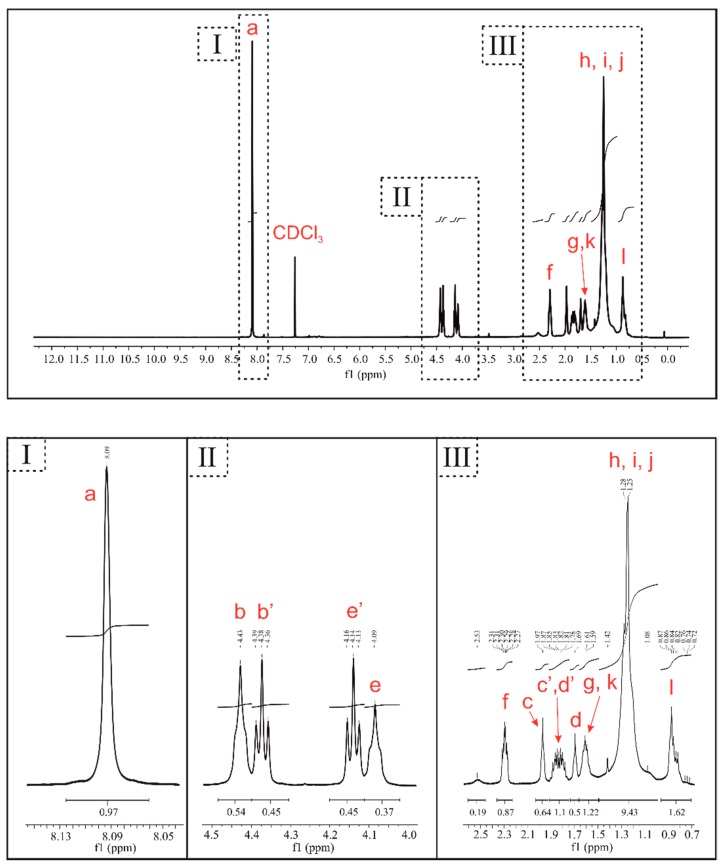
^1^H NMR spectra of PBT–DLA copolymer.

In order to confirm that we obtained the desired PBT–DLA copolymer structure, we used NMR spectroscopy. The detailed structural analysis of ^1^H NMR spectra of PBT–DLA copolymer, as well as peaks assignments, are presented in Figure 1 and Figure 2, respectively.

The first signal at 8.09 ppm (a) was assigned to the four symmetrical equivalent protons in the benzene ring present in hard segment residues of terephthalic acid. Different signals for the protons of the methylene groups in the butylenedioxy moieties were distinguished in the NMR spectrum, indicating the arrangement of segments. The two signal pairs (4.47–4.34 and 4.18–4.05 ppm) and signals at 2.02–1.67 ppm correspond to α protons (b, b’, e, e’) and β protons (c, c’, d, d’) of the discussed methylene groups, respectively. All of the possible arrangements of the segments are shown in Figure 1. Another identified multiplet at 2.34–2.25 ppm (f) was assigned to the α-protons of the methylene group of the fatty acid sequence, located next to the ester bond (the DLA molecule has four such protons). Finally, the three signals in the range of 1.66–1.55 (g, k), 1.45–1.0 (h, i, j), and 0.93–0.77 ppm (l) were attributed to protons of methylene groups and terminal methyl groups in the long alkyl (C_34_) DLA chain. 

**Figure 3:**
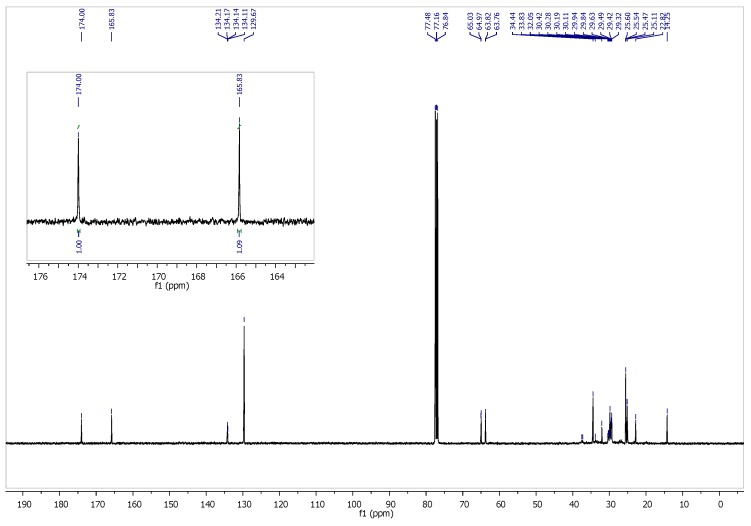
^13^C NMR spectra of PBT–DLA copolymer with a magnified region characteristic for carbons present in carbonyl groups.

Additionally, we confirmed the copolymer structure using ^13^C NMR (Figure 3). The analysis of two peaks at 174 ppm and 166 ppm (Figure 3 inset) corresponding to carbons in carbonyl groups in the neighborhood of aliphatic and aromatic structures, respectively, confirmed the segmental composition of PBT–DLA. The fraction of carbonyl groups surrounded by an aromatic ring, here 0.522, is in good correlation with the ^1^H NMR results (0.529). Taking into account the detection level of the ^13^C NMR method, as well as the coupling issues, it was more convenient to rely on ^1^H NMR for further analysis.

Based on the ^1^H NMR spectra, the degree of polycondensation of the hard segments in PBT–DLA copolymer was calculated and compared to the theoretical value of 1.21. Since the peak at 8.09 ppm is assigned to four protons in the benzene ring of PBT sequences and the peaks at 2.30 ppm arising from four equal protons in DLA sequence, DP_h_ was calculated from the following equation:

(1) $$DP_{h}=\frac{I_{8.09}}{I_{2.30}},$$

where DP_h_ is degree of polymerization of hard segments, I_8.09_ is the integral of peak at 8.09 ppm, and I_2.30_ is the integral of peak at 2.30 ppm. The weight percentage of hard segments (%W_h_) of PBT–DLA copolymer was calculated from the DP_h_ value using Equation (2):

(2) $$\%W_{h}=\frac{DP_{h}\cdot M_{H}}{DP_{h}\cdot M_{H}+M_{S}},$$

where %W_h_ is weight percentage of hard segments, DP_h_ is the degree of polymerization of hard segments, and M_H_ and M_S_ are the molecular weights of hard and soft segments, 220 and 620 g/mol, respectively. The results of proton NMR analysis of the PBT–DLA copolymer composition are presented in Table 2.

The molecular weight of the obtained PBT–DLA copolymer was determined using gel permeation chromatography (GPC). A sample of copolymer was dissolved in THF, filtered, and measured at 35 °C using polystyrene standards (5–1470 kDa). The results are presented in Table 2.

As can be seen in the results presented in Table 2, we obtained a very good match between the theoretical value of hard segment content (predicted from feed) and that calculated from proton NMR (30 vs. 28.5 wt%; 55.3 vs. 52.9 mol %). The actual DP_h_ value is lower (1.11) as compared to the theoretical one (1.21), because the yield of the first stage of the reaction was not 100%.

Further, based on the ^1^H NMR and GPC results, a more detailed analysis of the segmental distribution within the copolymer structure was performed. After integration of the peaks corresponding to the characteristic protons of methylene groups between two hard segments (HH, signal “c” (1.97 ppm)), two soft segments (SS, signal “d” (1.69 ppm)), and hard–soft segments (HS, SH, signals c’, d’ (1.83 ppm)), it was possible to determine the molecular architecture, including the average sequence length (*L_H_, L_S_*) of hard and soft segments, respectively, and the degree of randomness, *R*, using Equations (3)–(5) [50,51]:

(3) $$L_{H}=\frac{HH+0.5\left(HS+SH\right)}{0.5\left(HS+SH\right)},$$

(4) $$L_{S}=\frac{SS+0.5\left(SH+HS\right)}{0.5\left(SH+HS\right)},$$

(5) $$R=\frac{1}{L_{H}}+\frac{1}{L_{S}},$$

where HH is the integral of the c signal, SS is the integral of the d signal, and HS/SH is the overall integral of the c’ and d’ signals. The average sequence lengths of hard segments and soft segments were calculated to be 2.16 and 1.91, respectively, and, thus, the degree of randomness, *R* was calculated to be 0.99. This value is very close to 1, indicating a random distribution of segments within the macromolecule. Thus, the identity of a given segment is independent of that of the adjacent ones. Importantly, this does not mean that the copolymer has an alternating segmental composition; instead, the sequences of segments can be modeled as a series of independent binary combinations (hard segment vs. soft segment), known in probability as Bernoulli trials, that follow the binomial distribution [52]. Based on the segmental composition (derived from ^1^H NMR and confirmed by ^13^C NMR, Figure 3) and the number average molecular weight (obtained from GPC, 41000 g/mol), we calculated that an average PBT–DLA macromolecule has ~100 segments, according to the following formula:

(6) $$n=\frac{W_{H}\cdot M_{n}}{M_{H}}+\frac{W_{S}\cdot M_{n}}{M_{S}}.$$

This value can be considered to represent the number of Bernoulli trials, while the molar fraction of each segment represents the probability of that outcome. Thus, the expected number of hard segments can be calculated according to Equation (7):

(7) $$N_{H}=n\cdot p,$$

where n is a number of trials (here, number of segments, calculated from Equation (6), here 100) and p is the probability of hard segment occurrence (molar fraction of hard segments, determined from ^1^H NMR, here 0.529). Likewise, the number of runs of segments containing at least x hard segments can be computed as follows:

(8) $$N_{H_{x}}=n\cdot q\cdot p^{x},$$

where q is a probability of soft segment occurrence (1 − p) and x is the lower bound for the number of hard segments in succession. Using this formula, we calculated the number of runs of hard segments with lengths of 1+, 2+, 3+, etc. Next, by serial subtraction, we computed the expected number of segments with run of lengths of exactly 1, 2, 3, etc. The analogous calculations were also performed for soft segments. The results of the segment length distributions are presented in Table 3.

As can be observed, an average PBT–DLA macromolecule can be expected to have a run of hard segments at least as long as 6, and single hard segments represent only 22.4% of total hard segments, with the rest being in runs of 2 and longer. Likewise, single soft segments represent only 28.2% of total soft segments. Continuing the analysis, we also calculated the expected number of changes between segment (X) types:

(9) X=n·q·p.

From the binomial distribution analysis, we then estimated the number of switches between segment types to be 48.8 (2X–1), which compares favorably with 48.6, the value predicted from the ratio of integrated ^1^H NMR signal corresponding to hard–soft bonds to that of the total for all three bonds (Equation (10)):

(10) $$S_{\mathrm{NMR}}=\frac{I_{c^{\prime }d^{\prime }}}{I_{c}+I_{d}+I_{c^{\prime }d^{\prime }}}\cdot \left(n-1\right),$$

where S_NMR_ is a number of switches calculated from ^1^H NMR, I_c_, I_d_, and I_c’ d’_ are the integrals of corresponding peaks, and n is the number of blocks/segments in an average macromolecule. 

**Figure 4:**
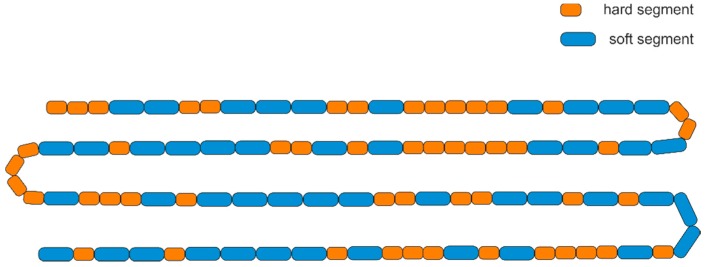
One possible arrangement of segments within an average PBT–DLA macromolecule.

Based on the above analysis, a possible structure of an average PBT–DLA macromolecule is presented in Figure 4.

From the standpoint of acting as a compatibilizer, it is important that the synthesized PBT–DLA copolymer, with a random architecture, is characterized by: (1) a high number of switches between segment types (butylene terephthalate vs. butylene dilinoleate) and (2) a range of lengths of each segment type (from 1 repeat up to 5 or 6). Thus, we anticipated that a sufficient number of “stitches” would be present at the interface and that at least some of the segments are of sufficient length to entangle with either of the two homopolymers in the blend.

### 3.2. Thermogravimetric Analysis (TGA)

**Figure 5:**
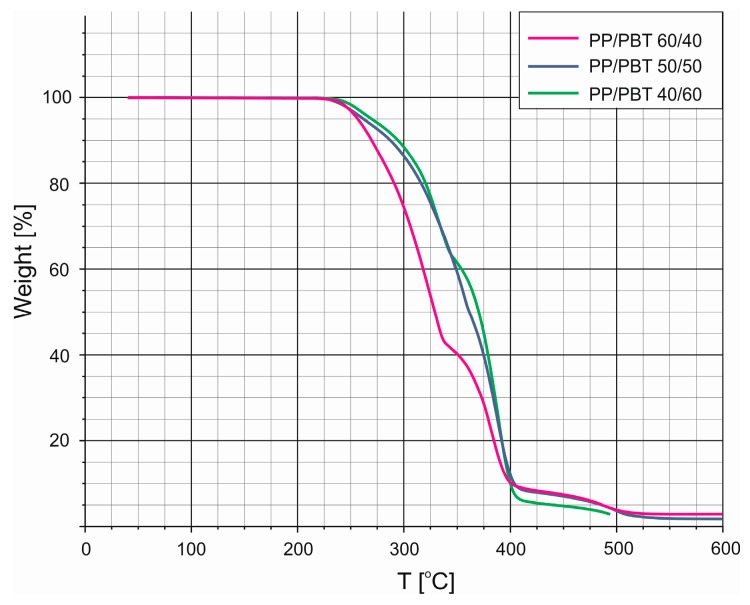
Non-isothermal analysis of TG thermograms of uncompatibilized polypropylene/poly(butylene terephthalate) (PP/PBT) blends.

The results of TGA carried out in air atmosphere for neat (uncompatibilized) PP/PBT blends are presented in Figure 5. Overall, the shapes of TG curves are very similar, and the residues left after incineration were very small. None of the blends exhibited any mass loss up to 220 °C, but at 250 °C, which is the standard processing temperature for PBT, 5% mass loss was observed for the blend containing the highest amount of PP (60/40). This is surprising, as other researchers have shown that PP is thermally stable, particularly under inert atmosphere, but even in the case of TGA in an air atmosphere, the *T*_5%_ is only reduced to 289 °C (vs. 384 °C under nitrogen) [53,54], which is considerably higher than what we observed for blends. In fact, even waste PP, after its life cycle, maintains superior thermal stability [55]. Further, neat PBT is also thermally stable, with no significant difference in thermal stability irrespective of the TGA measurement atmosphere, *T*_10%_ ~ 380 °C in air vs. 384 °C under argon [56,57]. In the light of this, such poor thermal stability of the uncompatibilized blends is most likely related to the lack of stability of the blend, which seems to be incompatible on a macroscopic level. We also observed that thermal stability of PP/PBT blends was significantly improved (by 50 °C at 50% of mass loss) for blends containing 50 and 60 wt% of PBT. This is a very important observation because 50% mass loss was noted for the 40/60 blend at 375 °C, while at the same temperature, substantially higher mass loss (up to 75%) was noted for the 60/40 blend. The TG curves also exhibit two regions with different slopes, signifying different decomposition rates, reflecting the two-component composition of the blends.

**Figure 6:**
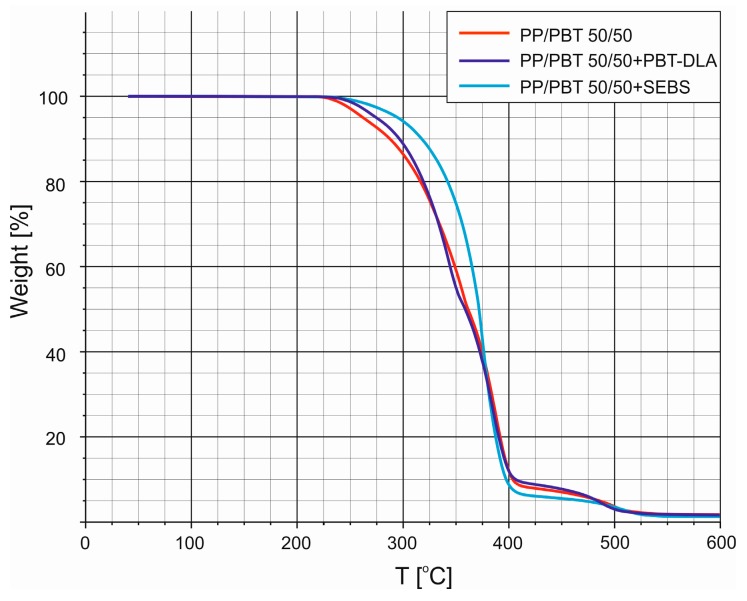
Non-isothermal analysis of TG thermograms of 50/50 PP/PBT blends with triblock (SEBS) and random (PBT–DLA) compatibilizers.

After the compatibilization process, all of the PP/PBT blends exhibited similar profiles of thermal decomposition. Thus, in order to better compare the blends with and without compatibilizers, we selected one composition (50/50) as a representative one (Figure 6). It should be highlighted that after addition of thermoplastic elastomers, the thermal stability of PP/PBT blends was enhanced, as evidenced by higher temperatures of initial decomposition, as well as the shape of the TG curve. Nevertheless, a greater effect was observed for the blend modified with triblock SEBS copolymer (one inflection of the curve) rather than with random PBT–DLA copolymer, which likely indicates greater homogeneity of this composition or higher thermal stability of SEBS.

**Figure 7:**
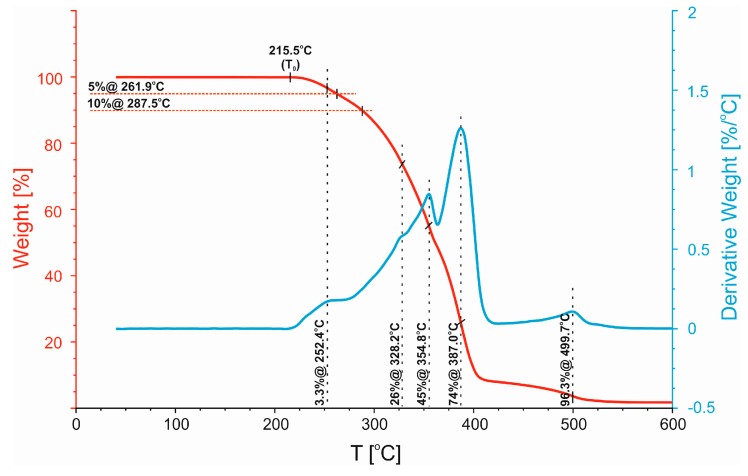
Non-isothermal analysis of TG and DTG thermograms of the uncompatibilized 50/50 PP/PBT blend.

**Figure 8:**
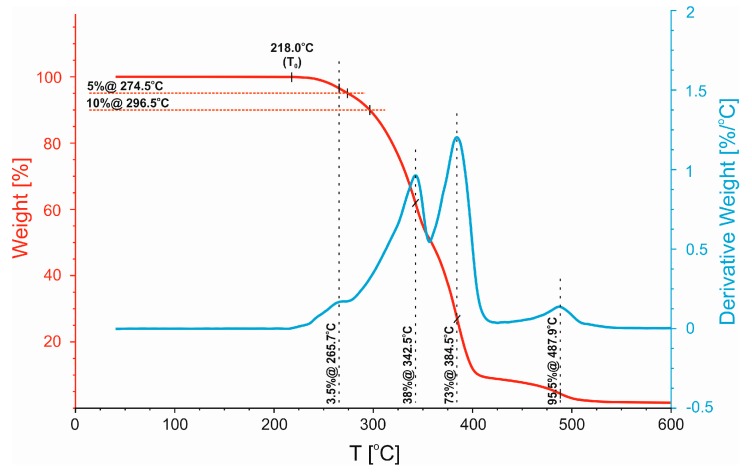
Non-isothermal analysis of TG and DTG thermograms of the 50/50 PP/PBT blend containing PBT–DLA copolymer.

**Figure 9:**
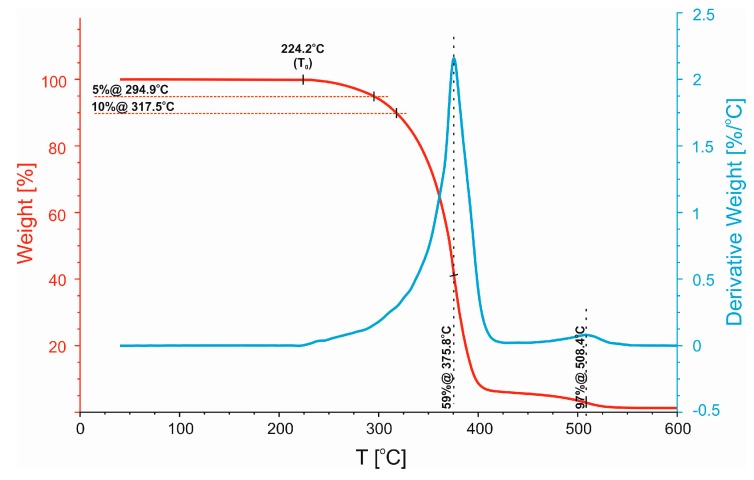
Non-isothermal analysis of TG and DTG thermograms of 50/50 PP/PBT blend containing SEBS copolymer.

Next, a more detailed analysis was used to describe the thermal properties of compatibilized and uncompatibilized 50/50 PP/PBT blends with respect to their thermal stability. Representative TG and differential TG (DTG) thermograms are presented in Figure 7, Figure 8 and Figure 9, along with a summary of the data in a tabular format in Table 4. For each blend, three different temperatures were compared: *T*_0_, temperature of initial thermal decomposition, where the thermal degradation begins, and *T*_5%,_ and *T*_10%_, temperatures of 5% and 10% mass loss, respectively. The shape of differential TG (DTG) thermograms was also compared. The uncompatibilized blend (Figure 7) exhibited relatively low temperatures of initial decomposition (*T*_0_ ~ 215 °C, *T*_5%_ ~ 262 °C, and *T*_10%_ ~ 288 °C). It is also characterized by a two-stage profile of the DTG curve. The first maximum (denoted as “45%@355°C”, see Figure 7) is related to decomposition of PP phase and the second one (denoted as “74%@387°C”), with the highest decomposition rate, is related to the decomposition of the PBT phase.

For the blend modified with PBT–DLA copolymer (Figure 8), the temperatures of initial decomposition are slightly higher (*T*_0_ ~ 218 °C, *T*_5%_ ~ 275 °C, and *T*_10%_ ~ 297 °C) (though the two-stage profile of the DTG curve remains essentially unchanged), but the decomposition of each phase peaks at lower temperatures compared to those for uncompatibilized blend. No additional signals that could be ascribed to PBT–DLA decomposition were noticed, indicating good dissolution/anchoring of the copolymer in the polymer blend.

The highest temperatures of initial thermal decomposition (*T*_0_ ~ 224 °C, *T*_5%_ ~ 295 °C, and *T*_10%_ ~ 317 °C) were observed for the blend modified with triblock SEBS copolymer (Figure 9). In this particular case, the DTG curve revealed a homogeneous profile, indicating greater blend homogeneity and improved ability of the individual components to interpenetrate each other across the well-developed interphase. As a result, with increasing temperature the individual phases decompose in parallel, at the same rate. Further, there are no visible signals indicating decomposition of the compatibilizer. 

### 3.3. Differential Scanning Calorimetry (DSC)

**Figure 10:**
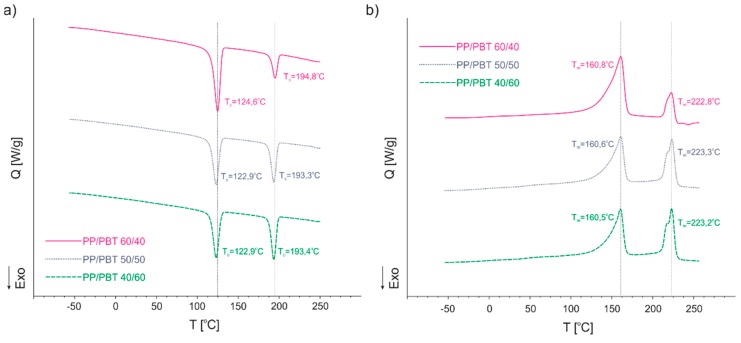
Differential scanning calorimetry (DSC) thermograms of uncompatibilized PP/PBT blends. (**a**) Comparison of crystallization behavior; (**b**) comparison of melting temperatures.

DSC analysis of polymer blends was performed in order to evaluate phase transition temperatures, as well as their thermal effects. The experiment was conducted in heating–cooling mode with the heating/cooling rate set to 10 °C/min. Because the thermal history of a polymer after processing (here, injection molding) affects the measured parameters, samples were tested after being subjected to a “thermal treatment” (first heating) to erase it. The DSC thermograms of uncompatibilized blends are presented in Figure 10, where both crystallization and melting behaviors can be observed.

**Figure 11:**
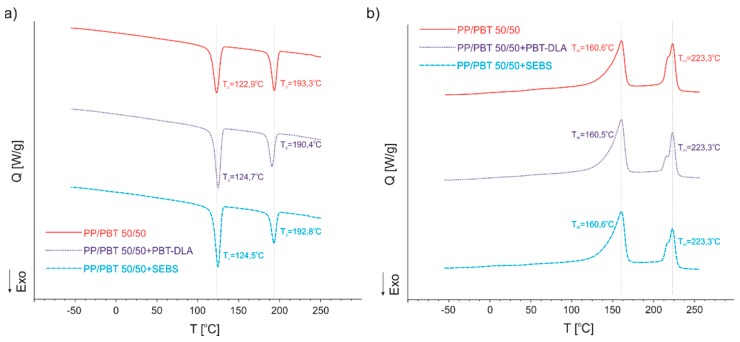
DSC thermograms of 50/50 PP/PBT blends with and without compatibilizers. (**a**) Comparison of crystallization behavior; (**b**) comparison of melting temperatures.

PP/PBT blends exhibited crystallization (maximum of exotherm) at different temperatures. The first one, at lower temperatures (123–124 °C), is ascribed to the PP, while the second one (at higher temperatures, 193–195 °C) is ascribed to the PBT phase. Only subtle differences in the crystallization temperatures were observed with change of the ratio of the components. In terms of the melting temperatures of uncompatibilized blends, no significant changes were observed (Figure 10b). Once again, in order to compare blends with and without compatibilizers, only one composition (50/50) was selected. The DSC thermograms of 50/50 uncompatibilized PP/PBT blend compared to blends modified with both types of compatibilizers are presented in Figure 11, while the numerical data are presented in Table 5.

Comparing blends with and without compatibilizers, one can observe changes in crystallization temperatures (T_c_): The crystallization temperature of PP has shifted towards higher temperatures, while for the PBT phase, the T_c_ has shifted towards lower temperatures. Such narrowing of phase transition temperatures, towards compatibilization, can be ascribed to the enhancement of blend miscibility after addition of compatibilizer [58]. Interestingly, this effect was more pronounced for the system compatibilized with random PBT–DLA copolymer, as can be observed by the greater shift in the T_c_ of the PBT phase. Further, for the blends with SEBS, we did not observe a decrease in the T_c_ of PP phase, as we had previously for blends prepared with PP and PBT homopolymers with markedly higher melt flow rates [40]. The addition of copolymers with different architectures did not have a significant effect on the melting temperatures (*T*_m_). Nevertheless, the blend modified with random PBT–DLA copolymer exhibited some changes in the shape of the thermogram (Figure 11b) in the region of PBT melting. Most likely, both of these changes are associated with the dissolution of a certain amount of PBT–DLA copolymer in the PBT phase of the blend. These observations are in good agreement with the theory that if a copolymer acting as blend compatibilizer has a more alternating structure, it can cover a broader part of the interface due to multiple passages between both polymeric phases [32,33,34,35,36]. In this case, this effect would be greater for the random PBT–DLA copolymer, with approx. 48 switches between segments, than in the case of triblock SEBS. 

**Figure 12:**
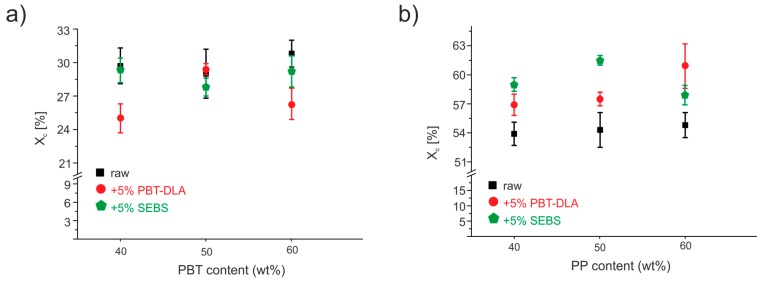
The effect of compatibilizer addition on degree of crystallinity of PP/PBT blends: (**a**) PBT phase; (**b**) PP phase. The data are presented as a mean of 3 measurements with standard deviations indicated by bars.

The degree of crystallinity, *X_c_*, of the polymer blends was determined through the measurement of the enthalpy of fusion and its normalization to the enthalpy of fusion of 100% crystalline polymer and calculated:

(11) $$X_{c_{1}}=\frac{\Delta H_{\mathrm{PBT}}}{\Delta H_{{\mathrm{PBT}}^{*}}\times W_{\mathrm{PBT}}}\times 100\%,$$

(12) $$X_{c_{2}}=\frac{\Delta H_{\mathrm{PP}}}{\Delta H_{{\mathrm{PP}}^{*}}\times W_{\mathrm{PP}}}\times 100\%,$$

where *ΔH_PBT_* and *ΔH_PP_* are the melting enthalpies of PBT and PP, respectively, measured with DSC, and *W_PBT_* and *W_PP_* are the mass fraction of PBT and PP, respectively. ΔH_PBT_* and ΔH_PP_*, which are the heat of fusion of PBT and PP with 100% crystallinity at equilibrium melting temperature, are 144.5 J·g^−1^ and 209.0 J·g^−1^, respectively, according to the literature [59,60]. The changes in degree of crystallinity of PP and PBT as a function of blend composition are presented in Figure 12a,b, respectively.

Regarding the crystallinity of the PP phase, an increase in crystallinity after addition of compatibilizers was observed (Figure 12b). It can be ascribed to changes in phase separation, due to compatibilization, and appearance of more space for PP crystals to grow. During cooling of the blend, the PBT crystals dispersed in the PP matrix act as heterogeneous nucleating agents and, thus, facilitate the crystallization of PP. The data also suggest that both compatibilizers were dissolved to a greater extent in the PBT phase, which is supported by the decrease in crystallinity of the PBT phase, especially for the blend with the highest amount of PBT, where a decrease from 31% for the uncompatibilized blend to 26% for the blend modified with PBT–DLA can be noticed (Figure 12a). Similar observations were noted in the work of Van Kets et al. [7], where, after addition of SEBS-*g*-MA to PP/PET systems, greater changes in the crystalline structure of PET were observed. The decrease in X_c_ of the PBT phase depends on the architecture of applied compatibilizer. The more alternating in structure, random PBT–DLA copolymer induced greater changes in crystallinity. This is likely due to the dissolution and potential multiple entanglements of a certain fraction of PBT–DLA within the outer layer of the PBT phase.

**Figure 13:**
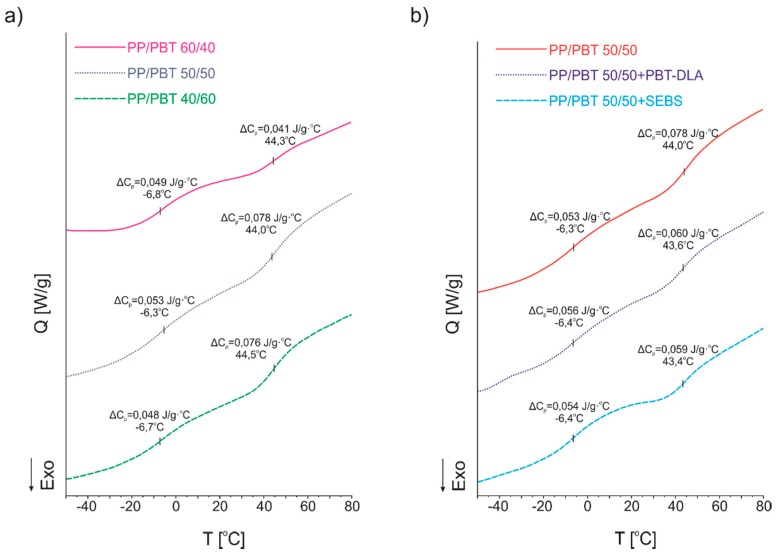
The effect of compatibilizers on glass transition temperatures of PP/PBT blends: (**a**) uncompatibilized blends with different mass ratio; (**b**) 50/50 blends before and after compatibilization.

Changes in the glass transition temperature (*T*_g_) can also be used as an indicator of compatibility of a binary polymer blend system. However, in the literature, there are some reports that DSC measurements for semicrystalline PP and PBT are not sensitive enough to detect glass transition [61]. In our case, the DSC method was sensitive enough to determine very minor changes in heat capacity (ΔC_p_) accompanying these transitions, with no change in *T*_g_ values (Figure 13; Table 5). No noticeable changes in the amorphous region were observed for blends with different composition, before and after compatibilization. However, the expected signals (at subzero temperatures) derived from the glassy phase of the highly elastic copolymer compatibilizers were also not detected using the DSC method. Overall, the heat capacity of the PBT phase (Figure 13b) for blends modified with both copolymers noticeably decreased. This observation is in good agreement with the changes in crystallinity, where the degree of crystallinity of the PBT phase also decreased. This may confirm the earlier assumption that the relative location of the compatibilizer within the blend depends on its molecular architecture. PBT–DLA is a random copolymer, with butylene terephthalate hard segments that, in principle, should have greater affinity for PBT and, thus, should be dissolved perfectly in the PBT matrix. However, the random structure should not allow it to penetrate deep enough into the PBT phase, thus preferentially locating it at the interface. On the other hand, the SEBS triblock copolymer has relatively long blocks (up to several kDa) and is able to dissolve in both phases simultaneously. Further, the grafting with maleic anhydride onto EB blocks provides miscibility with polar components, in this particular case with the PBT matrix, due to the formation of specific polar interactions between both components. It also enhances integration with the PP phase via van der Waals interactions between grafted EB blocks with polyolefin blend components.

### 3.4. Dynamic Thermomechanical Analysis

**Figure 14:**
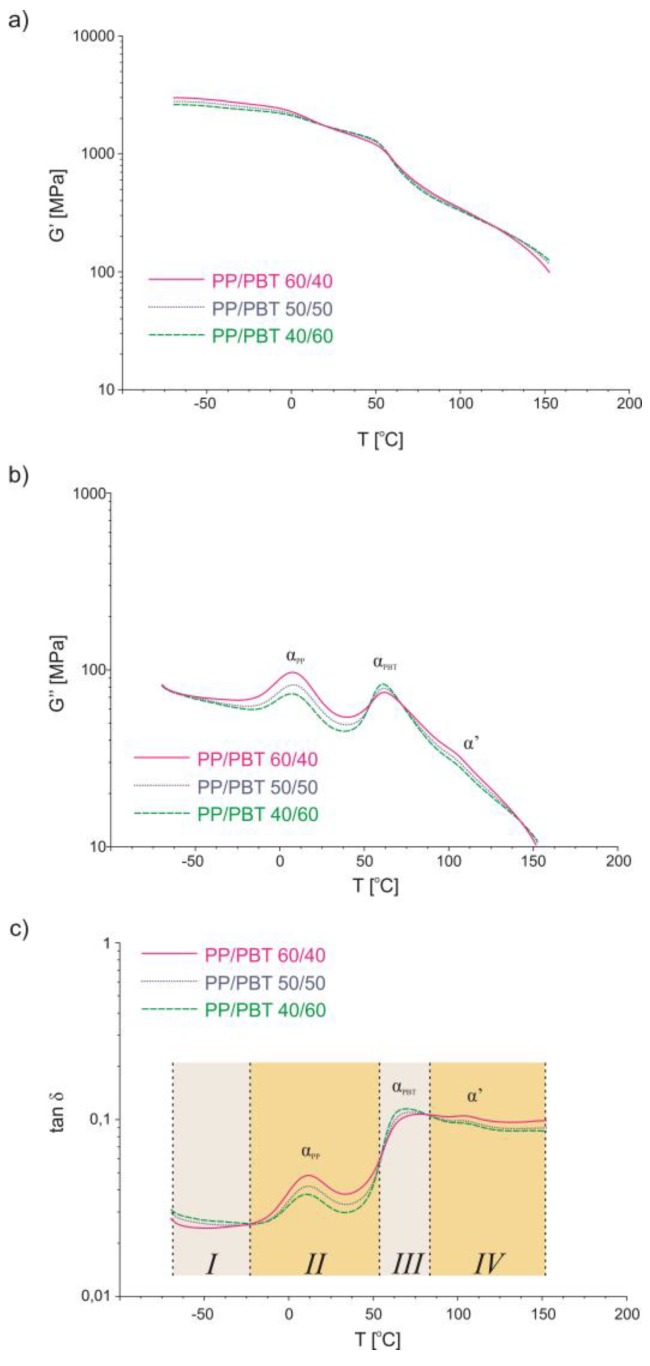
DMTA analysis of uncompatibilized PP/PBT blends: (**a**) storage modulus; (**b**) loss modulus; (**c**) tangent of delta.

**Figure 15:**
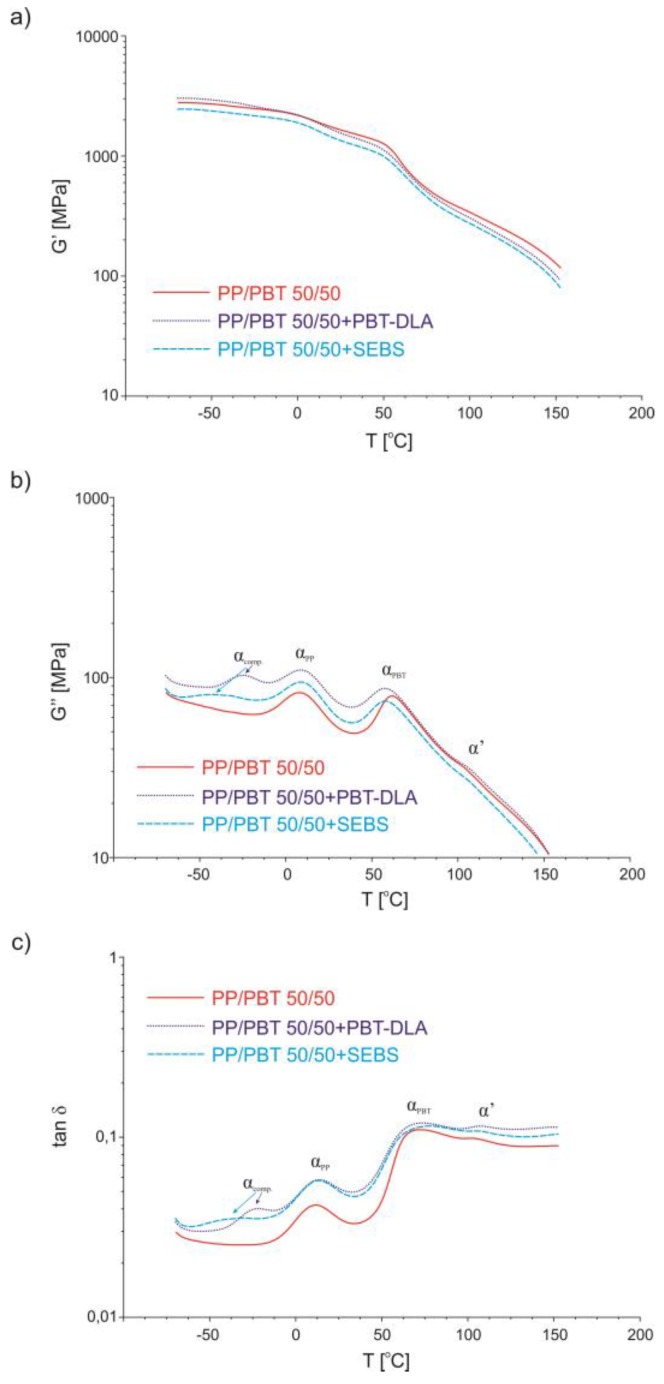
Dynamic thermomechanical analysis (DMTA) analysis of compatibilized PP/PBT blends: (**a**) storage modulus; (**b**) loss modulus; (**c**) tangent of delta.

Dynamic thermomechanical analysis (DMTA) was used to evaluate relaxation changes in the amorphous phase and to confirm the effectiveness of applied compatibilizers in enhancing blend miscibility depending on their molecular architecture. Miscible polymers exhibit thermodynamic solubility and should consist of a single phase, indicated by a single *T*_g_. Heterogeneous, immiscible blends exhibit phase separation, resulting in multiple *T*_g_’s of individual components. Complete immiscibility means that the *T*_g_’s of the individual phases are identical to the *T*_g_’s of the components [3]. On the other hand, if miscibility occurs, in the vast majority of polymer pairs, the *T*_g_’s shift closer to each other, towards compatibilization. The DMTA data for uncompatibilized PP/PBT blends of different compositions are presented in Figure 14. As previously, in order to compare blends after compatibilization, only one blend composition (50/50) was selected as a representative material. The values of the storage modulus, G’ (Figure 14a), representing elastic behavior in viscoelastic polymers, indicate that there are some slight changes in the elasticity with the change in blend composition, especially in the subzero temperature range—the PBT phase provides more elasticity in this region. After addition of compatibilizers (Figure 15a), the values of the storage modulus were slightly lower over the whole temperature range, indicating that elasticity increased, as expected. However, for the blend modified with PBT–DLA random copolymer, in the range of subzero temperatures, the opposite was observed, probably due to the glass transition of PBT–DLA taking place at this temperature range (onset ~0 °C; transition −25 °C). 

The shape of the loss modulus (G’’) curves (measure of viscous behavior in polymers), presented in Figure 14b and Figure 15b, shows two relaxation maxima (marked as α_PP_ and α_PBT_) corresponding to the amorphous phase of PP and PBT, respectively, and one additional feature (marked as α’) appearing at ~110 °C, before melting of PP crystals.

Thermograms of the loss modulus for blends after addition of compatibilizers (Figure 15b) revealed additional maxima corresponding to the amorphous phase of the compatibilizer (marked as α_comp._). In addition, a noticeable shift (approx. 5 °C) in the relaxation temperatures towards compatibilization was observed for blends modified with PBT–DLA and SEBS copolymers. As with the T_c_ changes, for the blends with SEBS, the change in PP phase relaxation is more subtle, as compared to our previous work with blends prepared with PP and PBT homopolymers with markedly higher melt flow rates [40].

The analysis of damping properties, here changes in tan δ (Figure 14c), revealed four distinct temperature regions for uncompatibilized blends. In the first region, at subzero temperatures, PBT damps more intensively, due to its elastic properties, and the highest values of tan δ were observed for the blend with the highest PBT content (40/60). In the next region, in the range of glass transition of PP, PP damps more effectively, due to its viscoelastic properties. Thus, here, the highest values of tan δ can be observed for the blend with the highest PP content (60/40). Next, in the temperature range in the neighborhood of the glass transition of PBT, PBT again damps more effectively, and the highest values of tan δ can be observed for the 40/60 blend. Finally, in the last region, above 100 °C, PP damps more effectively, probably due to the melting and recrystallization of PP in this region (again, the 60/40 blend reaches the highest value of tan δ). 

After compatibilization, damping capacity increased over the whole temperature range (Figure 15c). This is further evidence of enhanced miscibility between PP and PBT induced by both thermoplastic elastomers. By definition, damping (tan δ) quantifies the degree to which a material absorbs and safely disperses energy in the form of heat. Thus, a higher damping capacity of compatibilized PP/PBT blends can be associated with improved interfacial properties. Further, taking into account that material damping can also be defined as the volume of the material element in which the vibrational energy is dissipated, the observed effect can be attributed to the greater volume of the interphase. For both compatibilized blends, similar tan δ values indicate that a quantitatively comparable interphase was created, but detailed mechanisms of damping across the interphase are much more complex. Importantly, over the range of typical use temperatures (−20 to 100 °C), there are no noticeable differences in tan δ values between blends modified with either of the two copolymers, indicating a similar, positive effect on blend miscibility.

### 3.5. Mechanical Properties

The tensile properties (strength, modulus, and elongation) for PP/PBT blends before and after compatibilization are presented in Table 6. In the uncompatibilized blends, PBT acts as the continuous phase, with PP forming the dispersed phase; due to poor interfacial adhesion, Young’s modulus is dominated by PBT (Celanex 1600A; ~2550 MPa). The addition of highly elastic compatibilizers resulted in a pronounced reduction in Young’s modulus, as expected. Further, after compatibilization, we also observed a modest increase in tensile strength, indicating improved distribution of loads between phases. Importantly, both compatibilizers also resulted in a similar, marked increase in elongation at break. This effect can be attributed to improved miscibility and interfacial adhesion and predicts a more homogeneous morphology, with reduced phase separation. This was confirmed by the impact strength results (Table 6), showing an approx. 30% increase after compatibilization. Overall, we can conclude that both compatibilizers result in a similar reduction in brittleness, which can enable a wider range of applications. Further, the improvement in mechanical properties is more pronounced here, as compared to previously studied blends prepared with PP and PBT homopolymers with markedly higher melt flow rates [40,62]. Likewise, focusing on elongation at break, both compatibilized blends presented here reached somewhat higher values than those previously reported, such as blends compatibilized with PP-*g*-MA combined with a multifunctional epoxy resin (5–12%) [61] or ethylene-*co*-GMA (5–16%) [63]. 

### 3.6. Morphology

**Figure 16:**
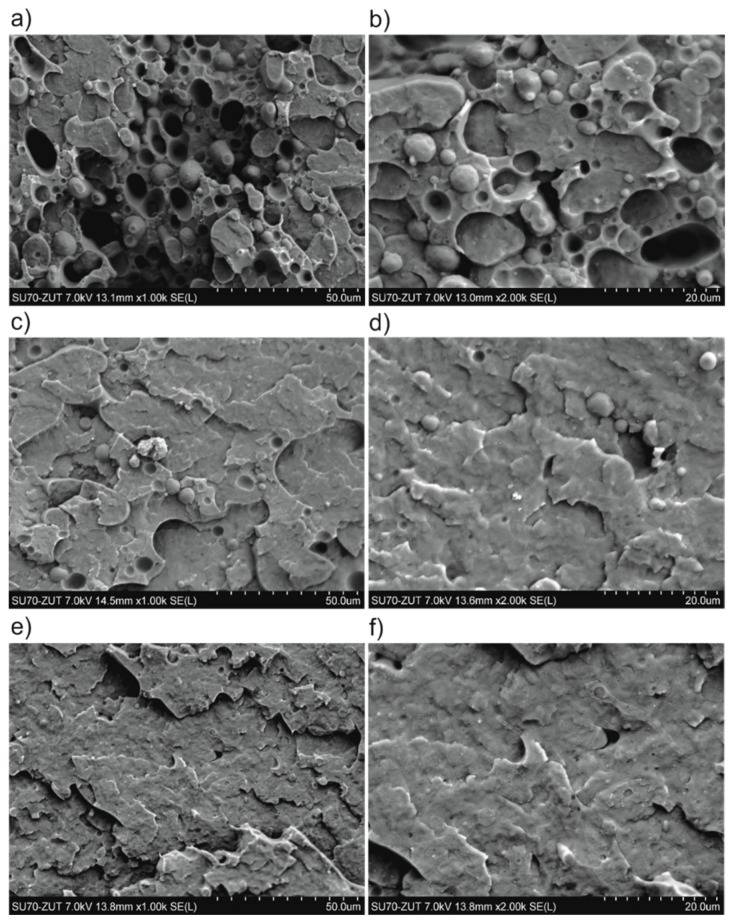
Micrographs of fracture surfaces of PP/PBT blends at different magnifications (x1000; x2000): (**a**,**b**) PP/PBT 50/50; (**c**,**d**) PP/PBT 50/50+5 wt% PBT–DLA; (**e**,**f**) PP/PBT 50/50+5 wt% SEBS.

The SEM micrographs of fracture surfaces (Figure 16) of representative 50/50 PP/PBT blends are the best direct evidence of changes in morphology after compatibilization. The blend without a compatibilizer exhibited strong phase separation. One phase, PP, is captured within a second one—the continuous PBT phase—forming spherical inclusions (Figure 16a,b), due to high interfacial tension, as a result of lack of compatibility and poor interphase. 

On the other hand, blends compatibilized with both copolymers (Figure 16c–f) were characterized by a comparable, more homogenous morphology—complete homogeneity between PP and PBT is thermodynamically unattainable, due to differences in chemical structure, molecular mass, as well as polarity. The triblock styrene copolymer (the well-known commercial compatibilizer), due to the high molecular mass of each block, is able to penetrate and dissolve in both the PP and PBT phases, bridging and “stapling” both phases together. As a result, domains are fused, and the boundaries between phases are blurred (Figure 16e,f). The random PBT–DLA copolymer, due to its different architecture (more numerous blocks of varied length), is distributed within the individual components in a quite different way. Nevertheless, this blend exhibited similar fracture morphology to that containing SEBS copolymer. This confirms that PBT–DLA copolymer is able to create a strong interphase between PP and PBT, with noticeably lower surface tension, interconnecting and “stitching” both phases. Here, the improvement in morphology is more pronounced than what we previously observed for blends prepared with PP and PBT homopolymers with markedly higher melt flow rates [62]. Importantly, the morphology of the blend prepared with PBT–DLA here (30/70 weight ratio) is markedly improved as compared to our previous work, where PBT–DLA 50/50 was used [44]. The weight ratio used here significantly shifts the distribution of the lengths of the butylene dilinoleate segments towards larger values, indicating that lengths greater than 3 are likely required to sufficiently interact with PP.

In the literature, several contradictory results can be found regarding the influence of copolymer architecture on the blend interphase. For example, Dadmun et al. demonstrated that random copolymers, with a tendency to crumple in on themselves, do not offer the opportunity for entanglements to occur, while architectures between random and block are able to penetrate along or across the interface and therefore significantly enhance the miscibility [30,31]. However, research on both polystyrene/poly(vinyl pyrrolidone)(PS/PVP) [64] and polystyrene/poly(methyl methacrylate)(PS/PMMA) [35,65] blends has shown that random copolymers can be very effective compatibilizers, due to crossing the interface multiple times, if the segments are long enough to entangle with the homopolymer. Importantly, in both of these cases, the compatibilizers were copolymers of the two constituents, which is somewhat analogous to our PBT–DLA copolymer in a PBT-containing blend. 

**Figure 17:**
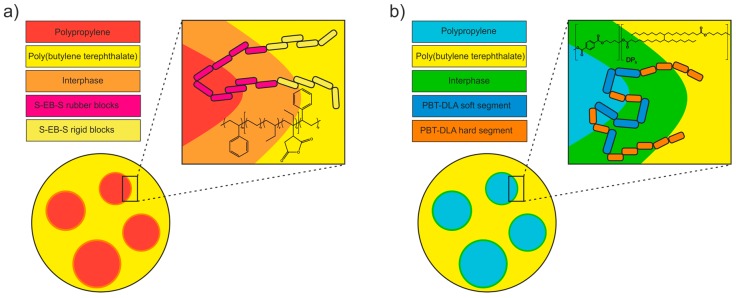
Probable compatibilization mechanisms using two different compatibilizers: (**a**) commercial triblock SEBS-*g*-MA copolymer; (**b**) polycondensation random PBT–DLA copolymer.

A graphical representation of the likely compatibilization mechanisms is presented in Figure 16. SEBS triblock copolymer grafted with maleic anhydride onto EB blocks has relatively long blocks, and each macromolecule crosses the interface twice, akin to a “staple” (Figure 17a). On the other hand, PBT–DLA, as a random copolymer, crosses the interface multiple times, preferentially locating it at the interface (Figure 17b). Further, the fact that the macromolecules have a range of segment lengths maximizes the chance of entanglement on either side. In this fashion, PBT–DLA should behave like “stitches”. Our thermal characterization strongly suggests that the butylene terephthalate hard segments are preferentially dissolved in the PBT phase, supporting the described mechanism.

## 4. Conclusions

Based on thermal stability, thermomechanical properties, static tensile and impact testing, and improved morphology, we concluded that PBT–DLA copolymer is suitable as a compatibilizer of PP/PBT blends. The TGA results indicated excellent thermal stability of blends compatibilized with both PBT–DLA and commercial SEBS copolymer. The melting behavior, crystallinity, and glass transition temperatures were affected in a similar way, despite differences in copolymer architecture. Importantly, static tensile and impact testing showed that both compatibilized blends were much less brittle. In particular, we noted an improvement in elongation at break to ~18% and ~21% for PBT–DLA and SEBS, respectively. Further, for blends compatibilized with the condensation copolymer, random PBT–DLA, a fine phase morphology was observed. This can be explained by the mechanism of the preferential location of short segments at the interface, crossing multiple times and “stitching” the two constituents together. We concluded that by using polycondensation, it is possible to obtain random copolymers containing monomers from renewable resources that act as efficient compatibilizers of polyolefin/polyester blends. In fact, the synthesized PBT–DLA copolymers can be 100% bio-based, making our findings particularly important, in light of the increasing awareness of sustainability in polymer science and engineering.

## Figures and Tables

**Table 1:** Selected physicochemical properties of SEBS copolymers.

Copolymer	*M*_n_ [kDa]	Density [g/cm^3^]	Tensile Strength [MPa]	MFI 230 °C, 5 kg [g/10 min]
SEBS	75–85	0.9	23–35	14–40

**Table 2:** Composition, degree of polymerization of hard segments and malar masses of PBT–DLA copolymer in comparison with commercial SEBS block copolymer [48,49].

Copolymer	Composition: wt% [mol %]		DP_h_		Molecular Weight			Dispersity Index
	Theoretical	Calculated	Theoretical	Calculated	[η] (dL·g^−1^)	*M*_n_ (g·mol^−1^)	*M*_w_ (g·mol^−1^)	D
PBT–DLA	30/70 [55.3/44.7]	28.5/71.4 [52.9/47.1]	1.21	1.11	0.907	41,000	115,000	2.8
SEBS	30/70 [48/52]	-	-	-	-	79,100/84,400	87,700/97,700	1.1/1.2

DP_h_—degree of polymerization of hard segments; [η]—intrinsic viscosity in phenol/trichloroethylene; *M*_n_—the number average molar mass; *M*_w_—the weight average molar mass, D—dispersity index (*M*_w_/*M*_n_).

**Table 3:** Segment length distributions.

Segment Length, x	# of Hard Segments at Least LENGTH x	# of Hard Segments Exactly Length x	# of Soft Segments at Least Length x	# of Soft Segments Exactly Length x
1	24.9	11.7	24.9	13.2
2	13.2	6.2	11.7	6.2
3	7.0	3.3	5.5	2.9
4	3.7	1.8	2.6	1.4
5	1.9	0.9	1.2	0.6
6	1.0	0.5	0.6	0.3
≥7	0.5	0.5	0.3	0.3

**Table 4:** Thermal decomposition temperatures of 50/50 PP/PBT blends.

Polymer Blend	*T*_0_ [°C]	*T*_5%_ [°C]	*T*_10%_ [°C]
50/50	215.5	261.9	287.5
50/50 + PBT–DLA	218.0	274.5	296.5
50/50 + SEBS	224.2	294.9	317.5

**Table 5:** Thermal properties of 50/50 PP/PBT blends.

Polymer Blend	*T*_c1_ [°C]	*T*_c2_ [°C]	*T*_m1_ [°C]	*T*_m2_ [°C]	*T*_g1_ [°C]	*T*_g2_ [°C]
50/50	122.9	193.3	160.6	223.3	−6.3	44.0
50/50 + PBT–DLA	124.7	190.4	160.5	223.3	−6.4	43.6
50/50 + SEBS	124.5	192.8	160.6	223.3	−6.4	43.4

*T*_c1_, *T*_c2_—crystallization temperatures of the PP and PBT phase, respectively; *T*_m1_, *T*_m2_—melting temperatures of the PP and PBT phase, respectively; *T*_g1_, *T*_g2_—glass transition temperature of the PP and PBT phase, respectively.

**Table 6:** Mechanical properties of 50/50 PP/PBT blends (mean ± SD, n = 7).

Polymer Blend	Young’s Modulus [MPa]	Tensile Strength [MPa]	Elongation at Break [%]	Impact Strength [kJ·m^−2^]
50/50	2130±250	35.2 ± 0.7	2.9 ± 0.2	4.1 ± 0.7
50/50 + PBT–DLA	1620±180	37.6 ± 0.5	18.2 ± 5.1	5.6 ± 0.3
50/50 + SEBS	1480±30	38.6 ± 0.3	21.2 ± 3.0	5.2 ± 0.4
